# Short communication: Performance evaluation of beef calves fed different levels of colostrum replacer in tropical conditions

**DOI:** 10.1016/j.vas.2026.100649

**Published:** 2026-04-08

**Authors:** João Paulo Ferreira Gomes, Giulia Berzoini Costa Leite, Marcos Inácio Marcondes, Jessica Marcela Vieira Pereira, Alex Lopes da Silva, Rafael Alves de Azevedo, Paula Marques Tiveron, Olayinka Miriam Tawose, Polyana Pizzi Rotta

**Affiliations:** a Department of Animal Science, Universidade Federal de Viçosa, 36570-000, Viçosa, Minas Gerais, Brazil; b Department of Animal Science, University of California, Davis, CA 95616-5270; c university Miner Agricultural Research Institute. Chazy, NY, 12921, USA; d Alta Brazil, Uberaba, Minas Gerais, Brazil

**Keywords:** Beef calf, Immunoglobulin G, Performance, Passive immunity

## Abstract

This study aimed to evaluate the effect of colostrum replacer supplementation on the health and performance of beef calves. Two studies were conducted to evaluate whether crossbred and purebred calves differ in their responses after receiving a colostrum replacer. In study 1, 162 ¾ Angus × Nellore calves born to ½ primiparous cows were assigned to three treatments: cow colostrum (no colostrum replacer: NCR; n = 54), cow colostrum + 50 g of immunoglobulin G supplementation (50IGG; *n* = 54), and cow colostrum + 100 g of IgG supplementation (100IGG, n = 54). In study 2, 244 Nellore calves were assigned to the same treatments: NCR (n = 82), 50IGG (*n* = 81), and 100IGG (*n* = 81). For both studies all calves had free access to cow colostrum. Serum protein concentrations were measured using a digital Brix refractometer. Calves were weighed using a tape at birth and weaning (80 days of age) to estimate average daily gain. In study 1, no significant differences (*P* > 0.0855) were observed among treatments for passive transfer. In study 2, significantly higher Brix concentrations were observed for calves supplemented with colostrum replacer (*P* = 0.01). No improvements on performance were observed for supplemented calves. These findings suggest that although all calves achieved successful transfer of passive immunity, colostrum replacer supplementation can further enhance passive immunity, as observed in Nellore calves. However, in production systems with adequate nutritional management, such as that used in the present study, colostrum replacer supplementation may not be necessary.

## Highlights

• Supplementing colostrum replacer increased serum Brix values in purebred Nellore calves but not in Angus × Nellore crossbred calves.
• All calves achieved successful passive transfer, indicating good colostrum management across treatments.
• In well-managed beef systems, additional colostrum replacer supplementation may offer limited performance benefits.

## 1 Introduction

In beef cattle operations, especially those under extensive management, calf handling within the first few hours of life is often delayed, increasing the risk of failure of transfer of passive immunity (FTPI) and early calf loss, and FTPI remains a major cause of early morbidity and mortality in beef calves (Raboisson et al., 2016; Ribeiro et al., 2007; Todd et al., 2018). Colostrum feeding is fundamental for the passive transfer of immunity from the dam to the calf (Godden et al., 2009). Colostrum is rich in immunoglobulins, essential for the neonatal immune defense, which is still immature at birth (Lopez & Heinrichs, 2022). These immunoglobulins play a crucial role in protecting the newborn against infectious agents during the neonatal period, thereby reducing the risk of morbidity and mortality (Weaver et al., 2000).

Although the importance of colostrum management and the use of colostrum replacers is well established in dairy calves (Cabral et al., 2013), research focusing on beef calves remains relatively limited. Existing studies have mainly characterized the prevalence and risk factors for FTPI in beef herds, but few have evaluated management interventions to prevent it (Elsohaby et al., 2019). This gap in the literature highlights the novelty and relevance of the present study. Moreover, beef cows managed under extensive or suboptimal environmental conditions may produce colostrum of inadequate quality or quantity (Souza, 2021), even when their nutritional requirements are met. Under these conditions, strategies that ensure adequate passive immunity transfer may play an important role in improving early calf health and survival in beef production systems. Furthermore, several factors influence the efficiency of immunoglobulin absorption by the calf, including timing of ingestion, volume consumed, and method of delivery, further emphasizing the need to explore alternatives such as colostrum replacers in beef herds (Godden et al., 2019; Teixeira et al., 2012).

Despite the established efficacy of colostrum replacers in dairy systems, their application in beef herds remains poorly studied, particularly regarding dose response and genetic background effects. We hypothesized that colostrum replacer supplementation improves passive immunity and early growth performance in beef calves. Thus, this study aimed to evaluate the effects of two supplementation levels of colostrum replacer on passive immunity transfer, health status, and performance of beef calves from different genetic groups.

## 2 Material and methods

The study was conducted on two commercial farms: Fazenda Santa Mônica (São João da Ponte, Minas Gerais, Brazil - 15° 55′ 44″ S, 44° 00′ 28″) and Fazenda Nelore Mocho CV (Presidente Venceslau, São Paulo, Brazil - 21° 53′ 19″ S, 51° 49′ 50″).

### 2.1 Study I

Study I was conducted using 204 calves (¾ Angus × Nellore), offspring exclusively from primiparous cows (½ Angus × Nellore), randomly distributed on three treatments: cow colostrum (no colostrum replacer: NCR; n = 68, 19 females and 35 males), cow colostrum + 50 g of immunoglobulin G supplementation (50IGG; n = 68, 21 females and 33 males), and cow colostrum + 100 g of IgG supplementation (100IGG, n = 68, 26 females and 28 males). Colostrum replacer was prepared at 40–45°C water and administered via esophageal tube until 12 hours of birth, following the manufacturer’s recommendation (Bovine Colostrum Replacer, The Saskatoon Colostrum Company). However, approximately 80% of administration was done until 4 hours after birth.

Calves were housed in maternity paddocks, into which dams were transferred 15 days prior to expected calving date. The cows received a total mixed ration composed of corn silage, grass silage, dried distillers grains, cornmeal, and a vitamin-mineral premix, with water provided ad libitum. The paddocks had natural shading provided by eucalyptus trees to ensure adequate animal welfare conditions and reduce stress. Only cows with a good body condition score were used in this study (5-6, in a 1-9 scale).

Blood samples were collected from all calves via jugular venipuncture using vacuum tubes containing clot activators within 24 to 48 hours post-colostrum replacer feeding. Blood samples were centrifuged at 2000 × g for 20 minutes at 4°C. Serum samples were subsequently separated, transferred to Eppendorf tubes, and stored at -20°C until analysis. Serum protein concentrations were determined using digital Brix refractometer (Instrument Choice Dry Creek).

Health monitoring was performed daily during the first 15 days, including visual assessment of diarrhea, pneumonia, and bovine parasitic sadness, based on McGuirk (2008). Diarrhea was evaluated by fecal consistency (firm, pasty, and watery), pneumonia by the presence and frequency of cough, nasal and ocular discharge, and ear positioning, and bovine parasitic disease (babesiosis/anaplasmosis) by ocular mucosa color and signs of apathy. All observed health incidents were documented promptly within the farm's health monitoring system, recording the date and specific diagnosis whenever applicable.

### 2.2 Study II

Study 2 was conducted using the same treatment design as study 1. A total of 244 Nellore calves were randomly allocated to one of three treatments: cow colostrum (no colostrum replacer: NCR; *n* = 81), 50 g IgG replacer (50IGG; *n* = 81), and 100 g IgG replacer (100IGG; *n* = 81). Colostrum replacer was prepared as described in Study I. Dams were moved to maternity paddocks 15 days prior to the expected calving date. During this period, cows grazed exclusively on pasture forage and had ad libitum access to water. Eucalyptus trees provided natural shading in the paddocks, improving animal welfare conditions and reducing stress.

Blood samples were collected from each calf between 24 and 48 hours after colostrum intake by jugular venipuncture, using vacuum tubes with clot activators. Serum was obtained by centrifugation at 2000 × g for 20 minutes at 4°C, separated into Eppendorf tubes, and stored at -20°C until analysis. Brix concentrations were measured using digital refractometer.

Calf body weight was estimated by using a girth tape (Nasco, Fort Atkinson, WI) within the first 24 h after birth and again at weaning (80 days of age) to calculate average daily gain (ADG). No health response could be recorded for this study.

### 2.3 Statistical analyses

Statistical analyses were performed using the GLIMMIX procedure of SAS University Edition (PROC GLIMMIX). The model included the fixed effects of treatment (0, 50, or 100 g of IgG) and sex, with initial body weight included as a covariate. The interaction between treatment and sex was tested and removed from the final model if not significant. As breed is confounded with study, separate analyses for studies 1 and 2 were performed, without a direct comparison between breed types. The significance level was set at *P* < 0.05, and tendencies were discussed when 0.05 ≤ *P* ≤ 0.10. When a significant treatment effect was detected, least square means were estimated.

## 3 Results and discussion

### 3.1 Study I

There was no significant influence of sex (*P* = 0.89) on Brix concentrations in calf serum, indicating that colostrum feeding efficiency was not affected by calf sex across treatments. However, a tendency was observed among treatments (P = 0.09; Table 1), with lower serum levels observed in the 50IgG and 100IgG treatments. Although a decrease in Brix values was observed, the values remained above the threshold considered adequate for passive transfer (≥ 8.9%), as established by Lombard et al. (2020), suggesting that all calves achieved effective passive immunity. According to Deelen et al. (2014), Brix percentage correlates closely with serum IgG concentrations, and values below 8.4% indicate insufficient passive immunity transfer. The adequate passive immunity observed in this study is crucial, as FTPI significantly increases morbidity and mortality risks in calves (Windeyer et al., 2014). Similarly, Silva et al. (2019) reported calves achieving success of transfer of passive immunity receiving maternal colostrum or colostrum replacers, aligning with the present findings. Additionally, Gamsjäger et al. (2023), in a study evaluating total and pathogen-specific serum IgG concentrations in neonatal beef calves and their associations with health and growth performance, reported that FTPI calves had higher risks of disease treatment and mortality, as well as reduced average daily gain during the pre-weaning period.

**Table 1:** Assessment of passive immunity transfer, and disease incidence in crossbred calves (Angus × Nellore) supplemented or not with colostrum replacer (Study I), and passive immunity transfer in Nellore calves supplemented or not with colostrum replacer (Study II).

Parameters	Sex		SEM	Colostrum			SEM	*P*-value		
	Female	Male		NCR	50IGG	100IGG		Sex	Colostrum	Sex × Colostrum
Study I										
Brix (%)	9.86	9.95	0.121	10.21	9.75	9.76	0.164	0.6472	0.0855	0.8946
Diarrhea (%)	1.29	1.38	0.224	1.29	1.44	1.27	0.294	0.8001	0.9197	0.8276
Pneumonia (%)	2.85	3.23	0.490	3.61	2.88	2.62	0.486	0.5743	0.5203	0.6315
Study II										
Brix (%)	10.22	10.07	0.20	9.90b	10.14ab	10.75a	0.20	0.079	0.0138	0.337

NCR – no colostrum replacer; 50IGG - 50 g of immunoglobulin G supplementation; 100IGG - 100 g of immunoglobulin G supplementation

No differences were observed among treatments for cases of diarrhea or pneumonia (*P* > 0.05; Table 1). However, previous studies have reported pre-weaning morbidity and mortality rates ranging from 5 to 10% in Brazilian beef herds. The absence of morbidity observed in the present study likely reflects the effectiveness of the overall management practices adopted, particularly those related to colostrum feeding. Although individual colostrum intake from the cow was not recorded, calves may have consumed adequate amounts to ensure sufficient passive transfer of immunity, which is essential to enhance disease resistance in newborns. This finding is especially noteworthy given that crossbred Angus × Nellore calves raised under extensive systems are often exposed to environmental stressors that can compromise passive immunity. Therefore, the favorable health outcomes observed here highlight the benefits of effective colostrum management in improving early calf health and survival in beef crossbred systems.

### 3.2 Study II

Brix concentration in calf serum was significantly affected by treatment (*P* = 0.01; Table 1), with higher values observed in calves supplemented with 50 or 100 g of IgG. The lower Brix values in serum recorded in non-supplemented calves may be attributed to factors such as poor colostrum quality, insufficient intake, or delayed ingestion (Godden et al., 2009). Because of the management characteristics of the beef production system, these variables could not be measured, which represents a limitation of the present study.

**Fig. 1:**
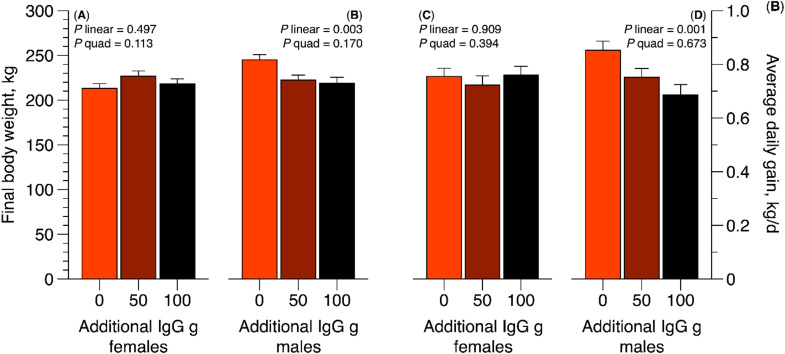
Final body weight (kg) and average daily gain (kg/d,) for Nellore supplemented or not with colostrum replacer by sex (Study II).

The initial bodyweight for this study was 31.3 ± 1.24 kg. An interaction between final body weight and ADG with sex were observed. For female calves no differences were observed for final body weight and average daily gain (Fig. 1). However, for male calves a linear effect was observed, were the NCR calves presented higher final body weight and average daily gain compared to the 50 and 100 IgG calves. This outcome may be associated with differences in management conditions during the pre-weaning period, such as variation in milk availability, nursing behavior, or access to feed and water.

Additionally, some level of cross-contamination could have occurred during colostrum feeding, which might have increased exposure to pathogens and contributed to higher disease incidence among supplemented calves (Cabral et al., 2013). This may have resulted from the use of shared feeding equipment, such as bottles, nipples, or feeding tubes, inadequate sanitation between feedings, or handling of colostrum during collection, storage, and delivery. Under these conditions, contamination with environmental or fecal bacteria could increase pathogen exposure in neonatal calves (Godden et al, 2019). However, disease occurrence by treatment were not recorded. The absence of systematic health monitoring represents a limitation of the present study, as it prevents a clear assessment of whether disease incidence differed among treatments. Consequently, the potential influence of health status or other unmeasured factors on growth performance cannot be excluded. Therefore, the greater growth observed in the NCR group may reflect the combined effect of individual cow performance, environmental variation, and unrecorded health events.

The contrasting outcomes observed between Study 1 and Study 2 suggest that the effectiveness of colostrum replacer supplementation may vary according to the genetic background of the calves. In Study 1, involving crossbred calves (¾ Angus × Nellore), supplementation with additional colostrum replacer did not improve passive immunity or growth performance, possibly due to enhanced neonatal vigor and more efficient absorption of maternal colostrum, benefits commonly associated with heterosis (Gregory et al., 1991, Schmidek et al., 2013). Conversely, in Study 2, purebred Nellore calves exhibited a significant increase in Brix concentrations in calf serum when supplemented with colostrum replacer. However, all calves achieved successful transfer of passive immunity. These findings reinforce the importance of adapting colostrum replacer use according to the system and management conditions, recognizing that colostrum supplementation may be more beneficial in beef herds facing challenges related to colostrum feeding.

## 4 Conclusions

The use of colostrum replacer supported adequate passive immunity transfer in both studies; however, the response was more pronounced in purebred Nellore calves, which showed increased Brix concentrations with supplementation. These findings suggest that, under adequate maternal nutrition and effective natural colostrum intake, the routine use of colostrum replacer may not be necessary for beef calves. This study contributes new insights to beef calf management by demonstrating that supplementation strategies should be tailored according to genetic background and production context.

## Ethical statement

All animal experimental procedures were performed in accordance with the ethical regulations regarding the use of animal specimen by the Ethics Committee on Animal Use of Universidade Federal de Viçosa (protocol number 108/2022), following established welfare guidelines. All efforts were made to minimize animal suffering and to reduce the number of animals used.

## CRediT authorship contribution statement

**João Paulo Ferreira Gomes:** Writing – review & editing, Writing – original draft, Project administration, Formal analysis. **Giulia Berzoini Costa Leite:** Project administration, Formal analysis. **Marcos Inácio Marcondes:** Writing – review & editing, Writing – original draft, Validation, Data curation. **Jessica Marcela Vieira Pereira:** Writing – review & editing. **Alex Lopes da Silva:** Validation. **Rafael Alves de Azevedo:** Writing – original draft, Methodology, Funding acquisition, Conceptualization. **Paula Marques Tiveron:** Methodology, Funding acquisition, Conceptualization. **Olayinka Miriam Tawose:** Writing – review & editing, Writing – original draft. **Polyana Pizzi Rotta:** Writing – review & editing, Writing – original draft, Visualization, Validation, Supervision, Project administration, Methodology, Formal analysis, Data curation, Conceptualization.

## Declaration of competing interest

The authors declare the following financial interests/personal relationships which may be considered as potential competing interests:

Polyana Pizzi Rotta reports financial support was provided by Federal University of Vicosa. If there are other authors, they declare that they have no known competing financial interests or personal relationships that could have appeared to influence the work reported in this paper.

## Data Availability

The data that support the findings of this study are available from the corresponding author upon reasonable request.

## References

[bib0001] Cabral R.G., Chapman C.E., Erickson P.S. (2013). Review: colostrum supplements and replacers for dairy calves. The Professional Animal Scientist.

[bib0002] Deelen S.M., Ollivett T.L., Haines D.M., Leslie K.E. (2014). Evaluation of a Brix refractometer to estimate serum immunoglobulin G concentration in neonatal dairy calves. Journal of Dairy Science.

[bib0003] Elsohaby I., McClure J.T., Cameron M., Keefe G.P. (2019). Evaluation of transmission of immunoglobulin G and associated calf-level factors in beef calves. Veterinary Record.

[bib0005] Gamsjäger L., Haines D.M., Lévy M., Pajor E.A., Campbell J.R., Windeyer M.C. (2023). Total and pathogen-specific serum immunoglobulin G concentrations in neonatal beef calves, part 2: associations with health and growth. Preventive Veterinary Medicine.

[bib0006] Godden S.M., Haines D.M., Hagman D. (2009). Improving passive transfer of immunoglobulins in calves. I: dose effect of feeding a commercial colostrum replacer. Journal of Dairy Science.

[bib0007] Godden S.M., Lombard J.E., Woolums A.R. (2019). Colostrum management for dairy calves. Veterinary Clinics of North America: Food Animal Practice.

[bib0008] Gregory K.E., Cundiff L.V., Koch R.M. (1991). Breed effects and heterosis in advanced generations of composite populations for preweaning traits of beef cattle. Journal of Animal Science.

[bib0010] Lombard J., Urie N., Garry F., Godden S., Quigley J., Earleywine T., McGuirk S., Moore D., Branan M., Chamorro M., Smith G., Shivley C., Catherman D., Haines D., Heinrichs A.J., James R., Maas J., Sterner K. (2020). Consensus recommendations on calf- and herd-level passive immunity in dairy calves in the United States. Journal of Dairy Science.

[bib0011] Lopez A.J., Heinrichs A.J. (2022). Invited review: the importance of colostrum in the newborn dairy calf. Journal of Dairy Science.

[bib0013] McGuirk S.M. (2008). Disease management of dairy calves and heifers. Veterinary Clinics of North America: Food Animal Practice.

[bib0014] Raboisson D., Trillat P., Cahuzac C. (2016). Failure of passive immune transfer in calves: A meta-analysis on the consequences and assessment of the economic impact. PLoS ONE.

[bib0015] Ribeiro A.R.B., Alencar M.M., Paranhos da Costa M.J.R., Negrão J.A. (2007). Effects of sire breed-grazing system and environmental parameters on the behaviour of beef calves just after birth. Applied Animal Behaviour Science.

[bib0016] Schmidek A., Paranhos da Costa M.J.R., Mercadante M.E.Z., Toledo L.M., Cyrillo J.N.S.G., Branco R.H. (2013). Genetic and non-genetic effects on calf vigor at birth and preweaning mortality in Nellore calves. Revista Brasileira de Zootecnia.

[bib0017] Silva A.P., Toledo A.F., Cesar A.M., Poczynek M., Coelho M.G., Silva M.D., Campos M., Bittar C.M.M (2019). Evaluation of passive transfer of calves receiving maternal colostrum or colostrum replacer. Journal of Dairy Science.

[bib0018] Souza L.A. (2021).

[bib0019] Teixeira W.T., Benesi F.J., Birgel Junior E.H., Mirandola R.M.S., Ayres M.C.C. (2012). Transfer of passive immunity and serum proteinogram in calves. Pesquisa Veterinária Brasileira.

[bib0020] Todd C.G., McGee M., Tiernan K., Crosson P., O’Riordan E., McClure J.T., Lorenz I., Earley B. (2018). An observational study on passive immunity in Irish suckler beef and dairy calves: tests for failure of passive transfer of immunity and associations with health and performance. Preventive Veterinary Medicine.

[bib0021] Weaver D.M., Tyler J.W., VanMetre D.C., Hostetler D.E., Barrington G.M. (2000). Passive transfer of colostral immunoglobulins in calves. Journal of Veterinary Internal Medicine.

[bib0022] Windeyer M.C., Leslie K.E., Godden S.M., Hodgins D.C., Lissemore K.D., LeBlanc S.J. (2014). Factors associated with morbidity, mortality, and growth of dairy heifer calves up to 3 months of age. Preventive Veterinary Medicine.

